# Development and psychometric evaluation of a postpartum stress scale in early postpartum mothers

**DOI:** 10.3389/fpubh.2026.1865506

**Published:** 2026-06-18

**Authors:** Dina Aprilia, Afra Hafny Noer, Retno Hanggarani Ninin, Zahrotur Rusyda Hinduan

**Affiliations:** 1 Faculty of Psychology, Universitas Padjadjaran, Bandung, Indonesia; 2 Faculty of Ushuluddin and Humanities, Universitas Islam Negeri Antasari Banjarmasin, Banjarmasin, Indonesia

**Keywords:** construct validity, postpartum mothers, postpartum stress, psychometric evaluation, scale development

## Abstract

The early postpartum period is a crucial phase in a mother’s adaptation to caregiving demands, physical recovery, and social pressures. In Indonesia, postpartum stress is further shaped by cultural norms, extended family involvement, and societal expectations. Despite the importance of these factors, measurement tools specifically tailored for the early postpartum phase in the local cultural context are limited. This study aims to develop a Postpartum Stress Scale to assess stress levels in early postpartum mothers, ensuring its construct validity and reliability. The research was conducted in two stages: the first stage utilized qualitative Focus Group Discussions (FGDs) with experts to establish construct and content validity, while the second stage employed Exploratory and Confirmatory Factor Analysis on data collected from 223 early postpartum mothers. The results indicated a robust four-factor structure with strong content validity and satisfactory psychometric properties, including high construct reliability and good model fit. The developed scale offers a reliable, multidimensional tool for assessing postpartum stress and holds promise for use in clinical practice and maternal mental health research.

## 1 Introduction

The postpartum period represents a critical transitional phase in a woman’s life, characterized by simultaneous biological, psychological, and social changes. During the early postpartum period, mothers are required to adapt to new roles as primary caregivers, recover physically after childbirth, and adjust to changes in family dynamics. These conditions place postpartum mothers at a heightened risk of experiencing psychological stress as a response to specific stressors associated with the puerperium, which may persist for up to 1 year after delivery (1). Empirical evidence indicates that the prevalence of postpartum stress ranges from 30 to 60% within the first 6 months following childbirth, with variations influenced by social, economic, and cultural factors (1, 2). Other studies report that approximately 40% of postpartum mothers experience moderate to high levels of stress, often associated with physical exhaustion, sleep disturbances, and inadequate social support (2). Postpartum stress not only affects maternal emotional well-being but also has significant implications for parenting quality, breastfeeding success, and long-term infant health and temperament (2).

Conceptually, postpartum stress is defined as a negative emotional experience arising from caregiving demands, changes in social roles, and environmental pressures accompanying the transition to motherhood (3). Contemporary psychopathological approaches position postpartum stress within a network of interacting psychological symptoms alongside depression and anxiety, where sleep disturbances and anhedonia serve as key bridging symptoms across mental health conditions (4). Furthermore, causal pathway studies demonstrate that postpartum stress has a direct effect on the development of depressive symptoms and functions as a mediator between marital satisfaction and maternal mental health (5). These findings highlight that postpartum stress is not merely a transient emotional reaction, but a complex psychological construct with significant clinical implications. In this context, the availability of a specific, valid, and reliable measurement instrument becomes essential. Existing instruments are predominantly generic or primarily focused on depression and anxiety, limiting their sensitivity in capturing the unique characteristics of postpartum stress, particularly in the early postpartum phase and within diverse cultural settings. Therefore, the development of a contextually grounded and psychometrically robust Postpartum Stress Scale is critically needed to enhance early detection accuracy, inform clinical interventions, and support evidence-based maternal health policies.

Based on previous studies, the measurement of postpartum stress is inherently multidimensional and requires contextual adaptation in its development to accurately capture the complexity of maternal experiences. Empirical evidence demonstrates that postpartum stress is influenced by various biopsychosocial factors, including biological conditions such as extreme fatigue, insomnia, and postpartum physical pain, which significantly contribute to increased psychological distress among postpartum mothers (6, 7). From a psychosocial perspective, low levels of social support during pregnancy and after childbirth have been consistently identified as major predictors of heightened parenting stress and emotional distress, whereas support from partners and family members, particularly maternal figures, functions as an important protective factor in reducing maternal stress levels (8–10). In addition, unmet psychosocial needs within maternal healthcare services, including limited emotional support and inadequate caregiving information, further increase the burden of postpartum stress (11, 12). Cultural factors also play a significant role, as social expectations regarding maternal roles, traditional postpartum practices, and family pressure in caregiving decision-making contribute to additional stress, especially in collectivistic societies (13). Pressure to exclusively breastfeed has also been associated with increased stress and decreased maternal well-being, particularly when mothers experience difficulties in the lactation process (14). Furthermore, traumatic childbirth experiences such as obstetric complications, emergency caesarean sections, and preterm births are recognized as major contributors to postpartum stress and may lead to more severe psychological conditions (15–17). These findings indicate that postpartum stress cannot be adequately captured using unidimensional or generic measurement tools, highlighting the importance of developing psychometric instruments that are multidimensional, context-sensitive, and aligned with the specific needs of postpartum populations.

Although research on postpartum mental health has grown rapidly, most studies continue to focus primarily on postpartum depression as the main indicator of maternal psychological disorders. Empirical evidence, however, indicates that stress represents a distinct psychological dimension with unique characteristics that should be measured separately from depression and anxiety (18). Postpartum stress also functions as a bridging symptom that connects clusters of depressive and anxiety symptoms, thereby playing a crucial role in the overall dynamics of maternal mental health disorders (19). Therefore, the specific measurement of postpartum stress is essential to obtain a more accurate understanding of mothers’ psychological conditions.

Various instruments have been developed to assess the psychological conditions of postpartum mothers, including the Edinburgh Postnatal Depression Scale (EPDS) (4, 5, 9, 20, 21), Perceived Stress Scale (PSS) (3, 9, 22), Parenting Stress Index (PSI) (8), Maternal Postpartum Stress Scale (MPSS) (1, 5, 6, 23, 24), Postpartum Specific Anxiety Scale (PSAS) (25–27), Hung Postpartum Stress Scale (28), Perceived Pressure to Breastfeed Scale (14), Perinatal Post-Traumatic Questionnaire (PPQ) (17), City Birth Trauma Scale (CityBiTS) (29), and Pandemic-Related Postpartum Stress Scale (PREPS-PP) (30, 31). However, most of these instruments are either generic in nature or primarily focused on specific mental health conditions, which limits their sensitivity in capturing the unique and context-specific stress experienced by mothers during the postpartum period (20, 32). The development of specialized scales such as the Maternal Postpartum Stress Scale (MPSS) demonstrates that postpartum stress possesses distinct characteristics that differ from general life stress and therefore requires dedicated measurement indicators (33). Cross-cultural validation of the MPSS further indicates high reliability and confirms that postpartum stress is a universal phenomenon, although it is shaped by variations in local cultural contexts (23). In addition, studies on the Postpartum Specific Anxiety Scale show that postpartum-specific instruments are more sensitive in detecting maternal distress compared to general psychological measures (25).

Although several postpartum-specific instruments have been developed, the literature indicates that significant limitations remain in the measurement of postpartum stress, particularly within the early postpartum context and among non-Western populations (21, 26, 27, 30, 31, 34). Most existing scales were constructed within different sociocultural contexts and primarily focused on selected psychological or adaptation-related domains, while some instruments concentrate on specific dimensions such as anxiety, childbirth trauma, or pandemic-related stress, limiting their ability to fully capture the holistic experience of maternal stress (26, 30, 31). Preliminary contextual exploration in Indonesian maternal settings suggested that postpartum stress experiences among Indonesian mothers are influenced not only by psychological distress, but also by collectivistic family dynamics, breastfeeding expectations, traditional postpartum beliefs, intergenerational caregiving involvement, and culturally shaped maternal role pressures. In addition, variations in population characteristics, such as adolescent mothers, mothers with preterm infants, and those with a history of mental health disorders, indicate that postpartum stress is inherently heterogeneous and requires measurement tools that are contextually grounded and sensitive to these diverse experiences (21, 27, 34). Therefore, the present study aimed to develop a culturally contextualized, multidimensional postpartum stress instrument rather than solely adapting existing scales.

In response to this gap, the development of a postpartum stress scale with strong construct validity and reliability becomes an essential need in the field of maternal health psychology. Psychometrically grounded instrument development enables a more objective and systematic assessment of postpartum stress, which can serve as a foundation for early detection, intervention planning, and evaluation of maternal health programs. Therefore, this study aims to develop and evaluate the psychometric properties of a postpartum stress scale for early postpartum mothers. This study is expected to contribute theoretically to the advancement of maternal mental health measurement tools and practically to the improvement of maternal healthcare services through a more comprehensive and evidence-based identification of postpartum stress.

## 2 Study 1

### 2.1 Methods

This study employed an exploratory approach consisting of two main stages, namely a literature review and a qualitative approach. The first stage involved a systematic literature review aimed at establishing a conceptual foundation, identifying the construct of postpartum stress, and comparing existing scales and instruments as references for indicator development. The literature review was conducted using the PRISMA approach by screening 40 relevant scientific articles, resulting in 26 articles that met the inclusion criteria and specifically addressed postpartum stress measurement and related instruments. The second stage applied a qualitative exploratory approach through Focus Group Discussions (FGDs) to identify and formulate the construct of postpartum stress in early postpartum mothers as the basis for developing scale indicators. This approach was intended to obtain an in-depth understanding of postpartum stress experiences from professionals with expertise in maternal health and perinatal psychology. The qualitative approach enabled the exploration of empirically and contextually relevant conceptual dimensions, ensuring strong content validity of the developed indicators. The integration of these two stages ensured that the instrument was grounded in both solid theoretical foundations and empirical evidence.

### 2.2 Participants

Participants in the qualitative stage consisted of five experts selected using purposive sampling based on their professional competence and experience in maternal and child health as well as maternal mental health. The inclusion criteria required participants to have relevant educational and professional backgrounds, experience in clinical practice, counseling services, or community empowerment programs related to postpartum mothers, and willingness to participate in the Focus Group Discussion (FGD). The participants included a psychologist with expertise in research methodology and maternal mental health, a lactation counselor experienced in supporting postpartum mothers, a midwife with experience in maternal and postpartum care services, a maternity nurse with experience in postpartum care, and a community or religious leader who plays a role in providing social and cultural support to postpartum mothers. The diversity of participants’ backgrounds was considered to obtain a multidisciplinary perspective in identifying postpartum stress experiences among early postpartum mothers.

### 2.3 Research procedure

**Figure 1:**
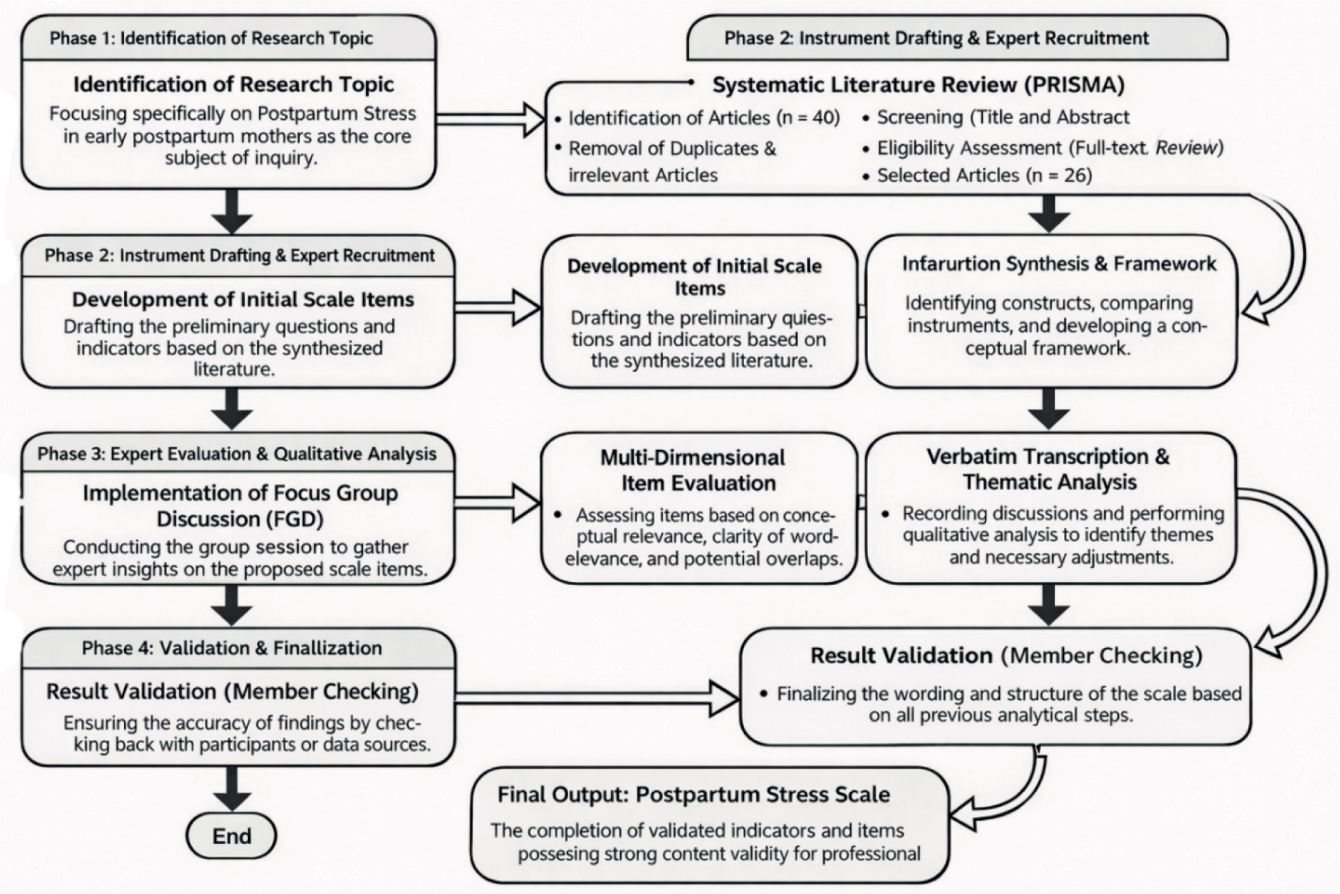
Flowchart of research procedure Study 1.

The research procedure began with the identification of the research topic focusing on postpartum stress in early postpartum mothers, followed by a systematic literature review using the PRISMA approach. A total of 40 articles were initially identified, then screened based on titles and abstracts, with duplicates and irrelevant studies removed. The remaining articles underwent full-text eligibility assessment, resulting in 26 selected articles. These articles were synthesized to identify key constructs, compare existing instruments, and develop a conceptual framework. Based on this synthesis, initial scale items were developed, followed by the preparation of a semi-structured Focus Group Discussion (FGD) guide. Expert participants (*n* = 5) were then recruited using purposive sampling. The FGD was conducted to evaluate the initial items in terms of conceptual relevance, clarity of wording, cultural appropriateness, and potential overlap between items. All discussions were recorded and transcribed verbatim for analysis. The data were analyzed using thematic analysis, followed by member checking to ensure the accuracy and credibility of the findings. The results were then used to revise and refine the items, leading to the final development of postpartum stress scale indicators and items with strong content validity. The flowchart of the research procedure for Study 1 is presented in Figure 1.

### 2.4 Data analysis

Data analysis began with the integration of findings from the Systematic Literature Review (SLR) conducted in the earlier stage to strengthen the conceptual foundation and identify key constructs of postpartum stress. The synthesized literature served as an initial framework to guide the interpretation of qualitative data and to compare emerging indicators. The data obtained from the Focus Group Discussion (FGD) were then analyzed using an inductive thematic analysis approach to identify conceptual themes representing the construct of postpartum stress. The analysis process involved repeated reading of transcripts to achieve a comprehensive understanding, coding meaningful units emerging from the discussion, grouping codes based on conceptual similarities, and interpreting the resulting themes into construct dimensions. The analysis also incorporated participants’ feedback regarding item clarity, relevance to postpartum experiences, and potential redundancy among items. In addition to thematic analysis, content validity was assessed using the Content Validity Index (CVI) to evaluate the relevance of each item based on expert panel ratings. This process included calculating the Item Content Validity Index (I-CVI) for each item and the Scale Content Validity Index (S-CVI) to assess overall expert agreement. The resulting themes, indicators, and CVI findings were subsequently used as the basis for revising and refining the postpartum stress scale items to be tested in the next stage of the study.

### 2.5 Results

The results of the systematic literature review indicate that the measurement of postpartum stress in previous studies has largely been dominated by partial instruments that focus on specific dimensions, such as depression, anxiety, or parenting stress. The synthesis of 26 selected articles revealed that the construct of postpartum stress generally encompasses psychological, physical, social, and contextual dimensions; however, these dimensions have not yet been comprehensively integrated into a single instrument specifically designed for the early postpartum period. Several existing instruments demonstrate limited sensitivity in capturing mothers’ experiences within specific cultural contexts and do not fully accommodate the dynamic interaction between biological, psychosocial, and cultural factors. These findings highlight the need for the development of multidimensional, contextually grounded, and empirically based instruments to enable a more accurate assessment of postpartum stress.

Study 1 aimed to identify the construct of early postpartum stress through conceptual exploration with an expert panel and to evaluate the content validity of the initial instrument items. The expert panel in Study 1 consisted of multidisciplinary professionals with expertise in maternal mental health, postpartum care, psychology, nursing, psychometric assessment, and maternal-child health research. The experts were selected based on their academic qualifications, clinical and research experience, involvement in maternal healthcare services, and expertise in postpartum psychological assessment and maternal mental health. The multidisciplinary expert evaluation process was intended to strengthen the conceptual, contextual, cultural, clinical, and psychometric appropriateness of the generated scale items. The results of the data analysis showed that most items were considered relevant by the experts; however, several items required refinement in wording and further conceptual clarification, particularly those related to physical fatigue, postpartum recovery experiences, and sociocultural pressures. The initial classification indicated that eight items were considered representative and required no further discussion, six items required wording revision, and seven items required deeper conceptual clarification, especially regarding potential overlap between indicators of postpartum physical experiences and concrete forms of family-related pressure experienced by mothers after childbirth.

The Focus Group Discussion (FGD) resulted in the refinement of item wording to enhance clarity, cultural relevance, and alignment with the clinical experiences of early postpartum mothers. Improvements included simplifying technical terminology into more operational language, clarifying the context of breastfeeding experiences, and strengthening the meaning of social pressure by incorporating concrete situations encountered by mothers. The discussion also emphasized that postpartum stress is not solely related to maternal role demands and infant caregiving, but is also influenced by postpartum physical conditions, family expectations, and the limited availability of social support during the early postpartum period.

Based on the synthesis of FGD findings, the construct of postpartum stress was formulated into four main dimensions: stress related to maternal role and infant care, physical stress and postpartum recovery, social and cultural pressure, and distress due to inadequate support. The first dimension reflects mothers’ experiences of uncertainty and anxiety in understanding their infant’s needs, concerns about breastfeeding adequacy, fear of making mistakes in caregiving, and perceived inability to fulfill the maternal role. The second dimension describes experiences of physical exhaustion, sleep disturbances, bodily discomfort, and limited rest following childbirth. The third dimension captures interpersonal and cultural pressures, including family comments, societal expectations regarding maternal roles, and traditional postpartum practices that may contribute to distress. The fourth dimension relates to experiences of insufficient support in infant care, limited partner involvement, and feelings of helplessness due to fatigue and lack of emotional and instrumental support.

Following the item revision process, content validity was assessed using the Content Validity Index (CVI). The results indicated that all 21 items achieved an I-CVI value of 1.00, suggesting that all experts rated each item as highly relevant to the construct being measured. Additionally, the S-CVI/Ave value of 1.00 reflects a very high level of agreement among experts regarding the overall representation of the postpartum stress construct within the developed instrument. These findings indicate that the items meet the criteria for content validity and are suitable for further empirical testing in a population of early postpartum mothers.

Overall, the results of Study 1 produced a set of instrument items that conceptually represent postpartum stress as a multidimensional and contextually grounded construct. The developed items not only capture mothers’ psychological experiences in adapting to their new roles but also incorporate physical, social, and cultural aspects that are distinctive to the early postpartum period. As a result, the instrument has the potential to demonstrate greater sensitivity in identifying postpartum stress compared to generic measurement tools. The key findings from Study 1 are summarized in Table 1.

**Table 1:** Summary of Study 1 results.

Category	Aspect	Summary of findings
Literature review (SLR)	Conceptual synthesis	26 selected articles indicate that postpartum stress is multidimensional (psychological, physical, social, contextual) but not yet integrated into a single instrument specific to the early postpartum period
Item development and evaluation	Initial item evaluation	21 initial items: 8 representative, 6 required wording revision, 7 required further conceptual clarification
	FGD results	Refinement of wording, clarification of breastfeeding context, inclusion of concrete social pressure situations, and improved cultural and clinical relevance
Construct development	Construct dimensions	Four main dimensions: (1) Maternal Role and Infant Care (2) Physical Stress and Recovery (3) Social and Cultural Pressure (4) Distress Due to Inadequate Support
	Construct characteristics	Postpartum stress is multidimensional and influenced by biological, psychological, social, and cultural factors
Content validity	CVI results	I-CVI = 1.00 for all items; S-CVI/Ave = 1.00 indicating very high expert agreement
Final output	Final instrument	21 items with excellent content validity, ready for empirical testing in the next stage

## 3 Study 2

Study 2 aimed to develop and empirically test the Postpartum Stress Scale through an item construction process based on the results of conceptual exploration and content validation conducted in Study 1. This stage produced a set of items representing the dimensions of postpartum stress, which were subsequently tested to obtain evidence of construct validity and instrument reliability.

### 3.1 Stage 1. Item construction

The item construction stage was carried out based on the results of conceptual exploration and content validity evaluation from Study 1, which generated operational indicators of postpartum stress in early postpartum mothers. The purpose of this stage was to develop a set of statements that comprehensively and measurably represent the construct of postpartum stress, referring to the conceptual framework of the Hung Postpartum Stress Scale, which defines postpartum stress as a negative emotional response experienced by mothers after childbirth, including feelings of being overwhelmed, pressured, anxious, fearful, lacking confidence, and experiencing sociocultural pressure (41). Operationally, postpartum stress in this study is defined as the level of emotional distress experienced by mothers after childbirth, reflected through four main dimensions: stress related to the maternal role as a new mother, physical fatigue and postpartum recovery, social and cultural pressure, and distress due to inadequate support. This variable focuses on mothers’ psychological responses to postpartum experiences rather than directly measuring the level of social support received.

Based on the synthesis of Focus Group Discussion findings, literature review, and content validity evaluation, a total of 21 items were identified as representing the construct of postpartum stress. The first dimension, maternal role and infant care stress, consists of seven items describing mothers’ uncertainty in understanding their baby’s needs, concerns about breastfeeding adequacy, fear of making mistakes in caregiving, and perceived inability to fulfill the maternal role. The second dimension, physical stress and postpartum recovery, consists of five items reflecting experiences of sleep disturbances, physical fatigue, bodily discomfort, and limited rest following childbirth. The third dimension, social and cultural pressure, consists of five items describing interpersonal pressure from family, societal expectations regarding maternal roles, and the influence of traditional postpartum practices that may cause psychological distress. The fourth dimension, distress due to inadequate support, consists of four items reflecting experiences of insufficient assistance in infant care, limited partner involvement, and feelings of helplessness due to fatigue and lack of emotional support.

All items were formulated as reflective statements describing the subjective experiences of early postpartum mothers. The wording of the items was designed to be simple, specific, and contextually relevant to ensure respondent comprehension and minimize ambiguity. In addition, each item was constructed to measure a single specific indicator in order to avoid double-barreled statements and enhance the psychometric quality of the instrument. The set of items developed at this stage was then used as the initial item pool for empirical testing in the scale validation phase.

### 3.2 Stage 2. Factor analysis

#### 3.2.1 Participants

Participants in the factor analysis stage consisted of 223 early postpartum mothers who were in the postpartum period and met the inclusion criteria for this study. The sample size was considered adequate for factor analysis as it satisfied the recommended respondent-to-item ratio, ensuring sufficient statistical power for Exploratory and Confirmatory Factor Analysis. The inclusion criteria for participants are outlined in Table 2.

**Table 2:** Inclusion criteria of participants.

No	Criteria
1	Mothers in the early postpartum period (within the first weeks after childbirth)
2	Aged 18 years or older
3	Able to read and understand the research instrument
4	Physically and psychologically stable at the time of data collection
5	Willing to participate voluntarily and provide informed consent
6	Recruited from maternal healthcare facilities (community health centers, maternity clinics, and hospitals) to ensure variability in respondent characteristics

Participants in Study 2 consisted of postpartum mothers with infants aged 0–6 weeks recruited from Indonesian maternal healthcare settings. Inclusion criteria included mothers who were able to communicate effectively, understand the questionnaire items, and voluntarily participate in the study. Exclusion criteria included severe medical complications, severe psychiatric conditions, cognitive impairment, or other conditions that could interfere with completion and interpretation of the postpartum stress assessment. Restricting participation to mothers within the first 0–6 weeks postpartum was intended to improve sample homogeneity while maintaining contextual representativeness of postpartum experiences among Indonesian mothers.

A detailed description of participant demographic and obstetric characteristics, including maternal age, postpartum period, parity status, educational level, and mode of delivery, is presented in Table 3 to improve transparency and contextual interpretation of the validation sample.

**Table 3:** Demographic and obstetric characteristics of participants (*N* = 223).

Demographic factors	*F*	%
Maternal age		
18–25 years	132	59.2
26–35 years	82	36.8
>35 years	9	4.0
Educational level		
Elementary school (SD)	3	1.3
Junior high school (SMP)	12	5.4
Senior high school (SMA)	113	50.7
Bachelor degree (S1)	82	36.8
Master degree (S2)	13	5.8
Household structure		
Nuclear family	148	66.4
Extended family	75	33.6
Parity status		
Primiparous	176	78.9
Multiparous	47	21.1
Mode of delivery		
Vaginal delivery	162	72.6
Caesarean section	61	27.4
Postpartum period		
≤1 week	25	11.2
2 weeks	58	26.0
3 weeks	36	16.1
4 weeks	37	16.6
5 weeks	37	16.6
6 weeks	30	13.5
Breastfeeding status		
Exclusive breastfeeding	193	86.5
Not breastfeeding	9	4.0
Mixed feeding	21	9.4

Inclusion criteria included postpartum mothers aged 18–40 years within 0–6 weeks postpartum who were able to communicate effectively, understand the questionnaire items, and voluntarily participate in the study. Exclusion criteria included severe medical complications, severe psychiatric disorders, cognitive impairment, or conditions interfering with questionnaire completion.

#### 3.2.2 Measurement

Measurement was conducted using the Postpartum Stress Scale developed during the item construction phase, consisting of 21 items representing four dimensions of postpartum stress: stress related to maternal role and infant care, physical stress and postpartum recovery, social and cultural pressure, and distress due to inadequate support. Each item was formulated as a reflective statement describing the subjective experiences of early postpartum mothers. Responses to each item were measured using a Likert scale indicating the degree to which respondents’ experiences aligned with the given statements, with higher scores reflecting higher levels of postpartum stress.

#### 3.2.3 Procedure

**Figure 2:**
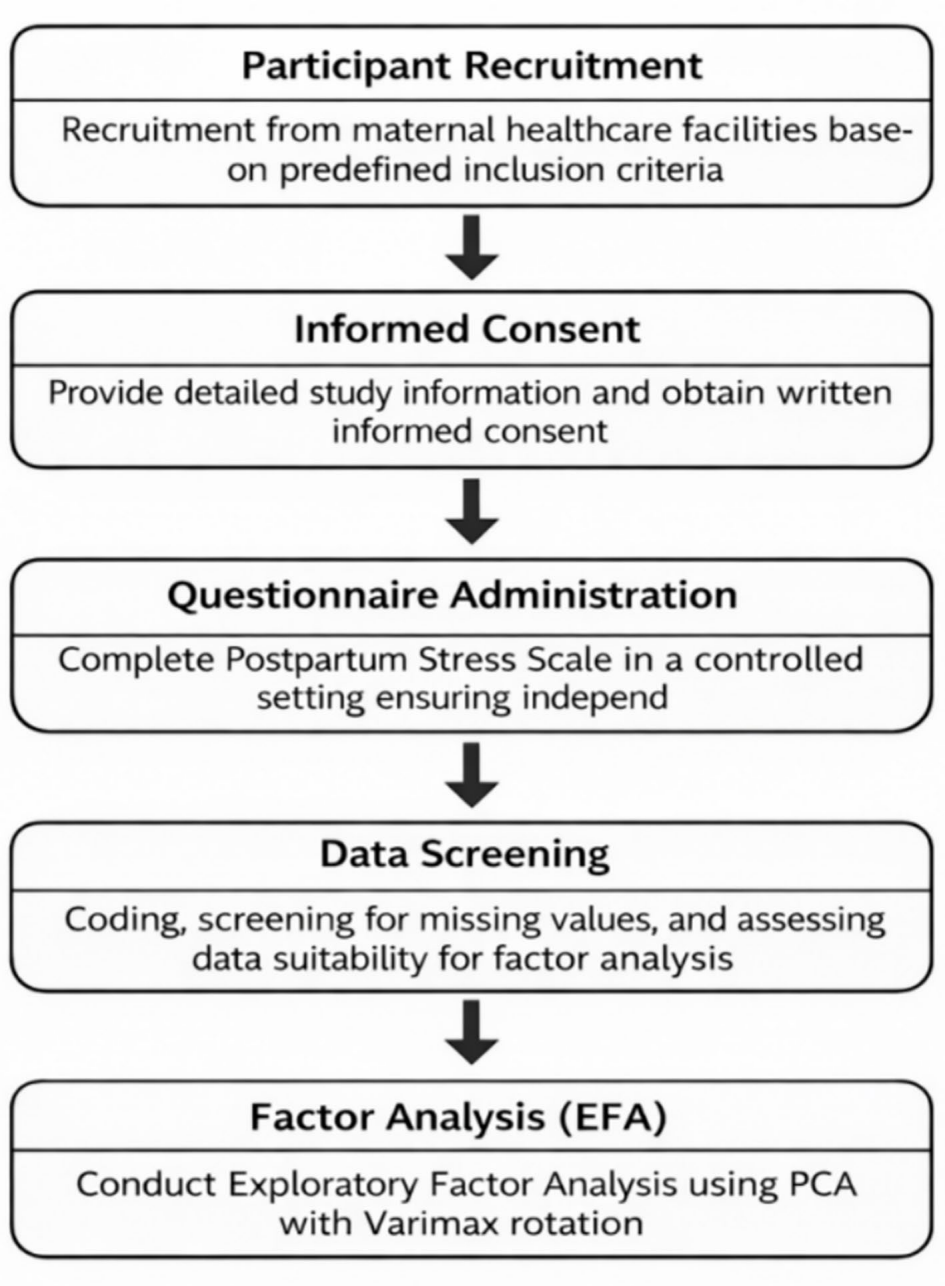
Flowchart of research procedure Study 2.

Data collection was conducted through a series of systematic stages involving participant recruitment, instrument administration, and data analysis. Eligible participants were recruited from maternal healthcare facilities, including community health centers, maternity clinics, and hospitals, based on predefined inclusion criteria. Participants were provided with detailed information regarding the study objectives, procedures for completing the instrument, and assurances of confidentiality and anonymity, after which written informed consent was obtained. Participants then completed the Postpartum Stress Scale consisting of 21 items in a controlled setting to ensure clarity of understanding and independent responses. The completed questionnaires were checked for completeness and accuracy before inclusion in the analysis. The data were subsequently prepared through coding, screening for missing values, and assessing suitability for factor analysis using the Kaiser–Meyer–Olkin (KMO) measure, Bartlett’s Test of Sphericity, and examination of the inter-item correlation matrix. Exploratory Factor Analysis (EFA) was then conducted using Principal Component Analysis (PCA) with Varimax rotation to identify the underlying factor structure, with the number of factors determined based on eigenvalues greater than one and interpretability. Factor loadings were evaluated to determine the contribution of each item to its respective factor. The results of the analysis were used to confirm the dimensional structure of the scale, guide item retention decisions, and refine the overall structure of the Postpartum Stress Scale prior to further validation. The research procedure for Study 2 is illustrated in Figure 2.

#### 3.2.4 Data analysis

Exploratory Factor Analysis (EFA) was conducted to identify the underlying factor structure of the postpartum stress scale and to evaluate the dimensionality of the construct. The analysis employed Principal Component Analysis (PCA) with Varimax rotation to achieve a clearer and more interpretable factor structure. Prior to factor extraction, data adequacy was assessed using the Kaiser–Meyer–Olkin (KMO) measure and Bartlett’s Test of Sphericity to ensure that the data were suitable for factor analysis. The number of factors was determined based on eigenvalues greater than one and the conceptual interpretability of the factor solution. Factor loadings were examined to assess the strength of the relationship between each item and its corresponding factor, with items retained based on meeting acceptable loading thresholds. The resulting factor structure was then interpreted to define the dimensions of postpartum stress and to support the construct validity of the developed scale.

#### 3.2.5 Results

The results of Study 1 led to the development of a preliminary Postpartum Stress Scale consisting of 21 items distributed across four dimensions derived from the integration of systematic literature review findings and expert evaluation through Focus Group Discussion (FGD). Each item was designed to reflect the subjective experiences of early postpartum mothers and to capture the multidimensional nature of postpartum stress, including psychological, physical, social, and support-related aspects. The items were formulated as reflective statements to ensure clarity, contextual relevance, and alignment with maternal experiences during the early postpartum period. The dimensions and items of the Postpartum Stress Scale are presented in Table 4.

**Table 4:** Dimensions and items of the postpartum stress scale.

Code	Dimension	Item
STRP1	Maternal role and infant care	I feel unsure how to calm my baby when he/she cries.
STRP2		I worry whether my baby is getting enough breast milk.
STRP3		I am afraid my baby might choke while breastfeeding.
STRP4		I am afraid of making mistakes in taking care of my baby.
STRP5		I feel overwhelmed by the many needs of my baby.
STRP6		I feel that I do not understand my baby’s needs.
STRP7		I feel that I am not capable of being a good mother.
STRP8	Physical stress and recovery	I am unable to get enough rest.
STRP9		I feel that my sleep is frequently disturbed or interrupted.
STRP10		I feel extremely physically exhausted.
STRP11		I experience bodily discomfort after childbirth.
STRP12		I feel that my body is sore and lacks energy.
STRP13	Social and cultural pressure	I feel disturbed by family comments about how I take care of my baby.
STRP14		I feel burdened by family expectations (parents, in-laws, and husband).
STRP15		Traditional beliefs during the postpartum period make me feel pressured.
STRP16		I feel that my family does not understand my condition after childbirth.
STRP17		I feel expected to handle everything on my own.
STRP18	Distress due to inadequate support	I feel burdened when I have to take care of my baby without help.
STRP19		I feel overwhelmed when my partner is less involved in baby care.
STRP20		Lack of attention from family makes me feel stressed.
STRP21		I feel helpless when I am exhausted and unable to rest.

The results of the data adequacy test showed a Kaiser–Meyer–Olkin (KMO) value of 0.943, indicating excellent sampling adequacy for factor analysis. In addition, Bartlett’s Test of Sphericity was significant (*p* < 0.001), suggesting that the inter-item correlation matrix was appropriate for factor analysis. The factor extraction results revealed the presence of four main factors with eigenvalues greater than one. These four factors cumulatively explained the variance of the postpartum stress construct. The Varimax rotation process produced a stable factor structure after six iterations. The four-factor structure explained a cumulative variance of 54.43%, indicating adequate explanatory power of the multidimensional postpartum stress construct and exceeding the commonly recommended minimum threshold of 50% for psychometric scale development.

The factor loading values indicated that all items loaded adequately on their respective factors. Items STRP1 to STRP7 showed the highest loadings on the first factor, items STRP8 to STRP12 on the second factor, items STRP13 to STRP17 on the third factor, and items STRP18 to STRP21 on the fourth factor. No items were found with factor loadings below the minimum acceptable threshold; therefore, all items were retained in the scale structure. The detailed factor loadings for each item across the four factors are presented in Table 1 and the rotated component matrix for the Postpartum Stress Scale is displayed in Table 5.

**Table 5:** Rotated component matrix of the postpartum stress scale.

Item	Factor 1	Factor 2	Factor 3	Factor 4
STRP1	0.859			
STRP2	0.825			
STRP3	0.803			
STRP4	0.763			
STRP5	0.785			
STRP6	0.779			
STRP7	0.834			
STRP8		0.777		
STRP9		0.783		
STRP10		0.791		
STRP11		0.628		
STRP12		0.730		
STRP13			0.798	
STRP14			0.872	
STRP15			0.824	
STRP16			0.812	
STRP17			0.847	
STRP18				0.847
STRP19				0.762
STRP20				0.802
STRP21				0.843

The results of the EFA were further examined using Confirmatory Factor Analysis (CFA) to evaluate the fit of the measurement model consisting of four dimensions. Model testing was conducted by comparing the four-factor model with an alternative one-factor model to assess the structure of the postpartum stress construct. The findings indicated that the four-factor model demonstrated a better model fit compared to the one-factor model. The fit indices for the Postpartum Stress measurement model are provided in Table 6.

**Table 6:** Fit indices of the postpartum stress measurement model.

Model	*χ*^2^ (df)	CFI	NNFI	SRMR	RMSEA
Four-factor model (21 items)	379.93 (185)	0.97	0.97	0.061	0.076
One-factor model	1,126.45 (189)	0.88	0.86	0.104	0.128

CFI, Comparative Fit Index; NNFI, Non-Normed Fit Index; SRMR, Standardized Root Mean Square Residual; RMSEA, Root Mean Square Error of Approximation.

**Figure 3:**
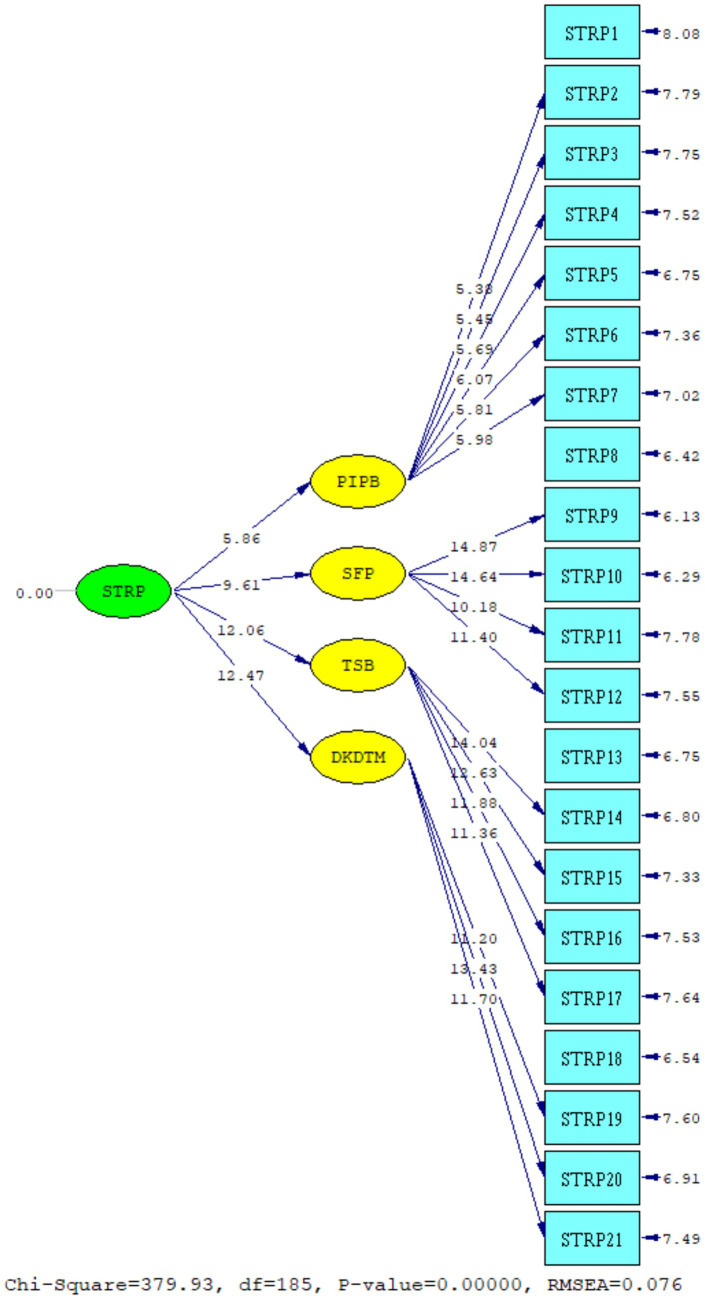
Confirmatory factor analysis model based on *t*-values.

Overall, the four-factor model met the criteria for incremental and residual fit indices, indicating that the multidimensional structure more accurately represents the postpartum stress construct. In contrast, the one-factor model showed a substantial decline in model fit, particularly in residual and approximation error indices. This difference suggests that a unidimensional approach is insufficient to capture the complexity of postpartum stress, which involves psychological, physical, and sociocultural aspects. Therefore, the CFA results support the four-dimensional structure as the most representative measurement model for postpartum stress in this study. The construct reliability results indicated that all factors demonstrated good internal consistency, with Composite Reliability (CR) values ranging from 0.852 to 0.867. In addition, convergent validity was achieved, with Average Variance Extracted (AVE) values ranging from 0.562 to 0.580, indicating that each factor adequately explains the variance of its indicators. The Confirmatory Factor Analysis model, based on T-values, is shown in Figure 3. Figure 3 presents the CFA T-value model used to assess parameter significance rather than standardized factor loadings. All standardized factor loadings were within acceptable psychometric ranges and supported the adequacy of the measurement model.

## 4 Discussion

The results of the Confirmatory Factor Analysis (CFA) indicate that all indicators demonstrated significant t-values in forming the postpartum stress construct. The t-values for each measurement path exceeded the statistical significance threshold, suggesting that all indicators contributed meaningfully to the formation of the latent construct of postpartum stress. These findings provide strong support for the construct validity of the developed scale and confirm that the multidimensional structure of postpartum stress is empirically robust.

The findings of this study are further strengthened by the results of the systematic literature review (SLR), which revealed that postpartum stress is a complex and multidimensional construct influenced by the interaction of psychological, physical, social, and cultural factors. The SLR highlighted that previous measurement approaches have largely relied on partial or domain-specific instruments, resulting in fragmented representations of postpartum stress. In contrast, the present study integrates these dimensions into a unified measurement model, thereby addressing the identified gap in the literature and providing a more comprehensive representation of postpartum stress, particularly in the early postpartum context. The results of this study demonstrate that postpartum stress is composed of four main factors that reflect the complexity of maternal psychological experiences following childbirth. This multidimensional structure is consistent with previous findings indicating that postpartum stress represents a negative emotional response to specific postpartum stressors and may persist for up to 1 year after childbirth (1). Postpartum stress is also closely associated with other psychological disorders, particularly depression and anxiety, where it functions as a bridging symptom within the network of maternal mental health conditions (4, 19, 35). These findings reinforce the importance of conceptualizing postpartum stress as a distinct yet interconnected psychological construct.

The first factor represents stress related to the transition into the maternal role and infant caregiving. This finding is consistent with prior research showing that uncertainty in understanding infant needs and the demands of caregiving are major sources of emotional distress among new mothers (3). Parenting-related stress is also associated with lower marital satisfaction and poorer interpersonal relationships, which may increase the risk of postpartum depression (5, 6). Furthermore, stress associated with the maternal role has direct implications for the quality of the mother–infant relationship and child development outcomes, including physical growth, breastfeeding quality, and long-term emotional bonding (2). Social support during pregnancy has also been shown to influence levels of parenting stress after childbirth (8). These findings emphasize that the transition to motherhood is a complex psychological adaptation process that requires emotional readiness and adequate social support. In line with the SLR findings, the integration of biological and psychosocial dimensions in this study highlights that postpartum stress is not solely a psychological phenomenon but also involves physical recovery processes and sociocultural dynamics. The multidimensional model developed in this study therefore offers a more holistic framework for understanding postpartum stress and provides a stronger empirical basis for measurement compared to existing instruments. Postpartum experiences among Indonesian mothers are strongly influenced by collectivistic family structures, traditional postpartum beliefs and practices, breastfeeding-related expectations, intergenerational caregiving involvement, and culturally shaped maternal role responsibilities. Therefore, the present instrument was developed to integrate psychological, physical, social, cultural, and support-related postpartum stress dimensions within a culturally contextualized psychometric framework relevant to Indonesian maternal settings.

The second factor indicates that postpartum stress is significantly influenced by physical conditions and the recovery process following childbirth. This finding reinforces the view that postpartum stress extends beyond a purely psychological phenomenon and is closely intertwined with maternal physical health, including extreme fatigue, sleep disturbances, and postpartum pain (6, 7). Previous studies have identified sleep disruption and physical exhaustion as central symptoms linking postpartum stress with depressive conditions (4). In addition, traumatic childbirth experiences further intensify postpartum stress, particularly among mothers who encounter obstetric complications or undergo medical interventions during delivery (15–17). These findings underscore the critical role of physical recovery as an essential component of maternal mental well-being during the postpartum period.

The third factor reflects the influence of social and cultural pressures on postpartum stress. Social expectations regarding maternal roles, family norms, and traditional postpartum practices can act as substantial sources of psychological strain. Cross-cultural evidence suggests that expectations from extended family members and adherence to traditional postpartum practices often increase stress, particularly when mothers experience limited autonomy in decision-making (13). Pressure to exclusively breastfeed may also contribute to elevated levels of emotional distress, especially when mothers face challenges during the lactation process (14). Furthermore, validation studies across different populations consistently identify social pressure and life role transitions as core components of postpartum stress (3, 28, 36). External contextual factors, including broader societal disruptions, may also intensify stress through heightened concerns about infant well-being and reduced social interaction (30, 31, 37).

The fourth factor highlights distress resulting from inadequate social support. This finding aligns with extensive evidence indicating that social support is one of the most important protective factors against postpartum stress (3, 9, 24). Support from partners, family members, and social networks plays a crucial role in reducing stress both directly and indirectly, particularly through enhancing marital satisfaction and strengthening mother–infant bonding. Meta-analytic findings further demonstrate that support from family members, especially maternal grandmothers, significantly reduces parenting-related stress in new mothers (10). Conversely, insufficient support increases vulnerability to postpartum mental health problems, including depression and Post-Traumatic Stress Disorder (PTSD) (21, 26, 34).

In addition, several studies emphasize the importance of distinguishing postpartum stress from perinatal depression. The development of perinatal-specific measurement tools demonstrates that emotional distress during the postpartum period possesses unique characteristics that are not fully captured by general depression scales (38). In the Indonesian context, postpartum stress is also associated with low maternal self-efficacy and the emergence of postpartum blues, indicating the importance of internal psychological processes in shaping maternal distress (39). Reviews of parenting stress instruments further reveal that existing measures remain predominantly focused on maternal populations, highlighting the need for more context-sensitive and specific assessment tools (40).

Emerging evidence from network analysis also suggests that certain postpartum stress symptoms act as bridging elements between depression and anxiety, particularly those related to sleep disturbances, lack of personal time, and perceived physical changes after childbirth (19). Social support influences postpartum stress through interpersonal mechanisms, including marital satisfaction and the quality of the mother–infant relationship (3). Moreover, clinical factors such as obstetric complications, severe fatigue, and emergency childbirth experiences have been identified as significant predictors of postpartum traumatic stress (29, 35). Postpartum stress is further shaped by internal cognitive processes, where negative cognitive reactivity can amplify stress responses and increase the likelihood of subsequent psychological disorders (22). Taken together, these findings confirm that postpartum stress is a multidimensional phenomenon resulting from the interaction of biological, psychological, and social factors, highlighting the importance of comprehensive and context-sensitive measurement approaches.

Postpartum experiences among Indonesian mothers are strongly influenced by collectivistic family structures, traditional postpartum beliefs and practices, breastfeeding-related expectations, intergenerational caregiving involvement, and culturally shaped maternal role responsibilities. Therefore, the present instrument was developed to integrate psychological, physical, social, cultural, and support-related postpartum stress dimensions within a culturally contextualized psychometric framework relevant to Indonesian maternal settings.

Compared with previously established postpartum stress instruments such as the MPSS and the Hung Postpartum Stress Scale (HPSS), the present scale demonstrated a different conceptual and dimensional approach in measuring postpartum stress. Previous instruments generally emphasized selected postpartum concerns and adaptation-related domains, including maternal role attainment, psychological adaptation, negative body changes, infant caregiving concerns, and lack of social support (33). In particular, the HPSS primarily organizes postpartum stress around concrete postpartum concerns and stressors, such as breastfeeding difficulties, infant health concerns, maternal overload, body image changes, and family-related stress experiences. Meanwhile, the MPSS mainly focuses on maternal psychological adaptation and emotional stress responses during the postpartum period. In contrast, the present scale was developed using a construct-oriented psychometric approach that conceptualizes postpartum stress as a multidimensional latent construct integrating psychological distress, physical recovery stress, sociocultural pressure, and support-related stress experiences within a single psychometric framework. The four-factor structure identified in this study consists of maternal role and infant care stress, physical stress and postpartum recovery, sociocultural pressure, and distress due to inadequate support. This multidimensional structure reflects the interaction between maternal recovery demands, caregiving adaptation, sociocultural expectations, and support-related stressors experienced during the early postpartum period. In particular, the sociocultural pressure dimension captures contextual stress experiences related to family expectations, traditional postpartum beliefs, interpersonal pressure, and culturally shaped maternal role demands commonly experienced within collectivistic maternal settings. In addition, the inadequate support dimension reflects not only practical assistance deficits, but also emotional distress associated with limited partner and family involvement during postpartum recovery and infant care. Therefore, the contribution of the present instrument lies not in claiming psychometric superiority over existing scales, but in extending the multidimensional and contextual representation of postpartum stress experiences within Indonesian maternal settings.

Compared with previously established postpartum stress instruments such as the Hung Postpartum Stress Scale, the present scale demonstrated satisfactory psychometric performance while integrating sociocultural and family-related postpartum stress experiences within a culturally contextualized framework relevant to Indonesian postpartum mothers. The contribution of the present instrument lies not in claiming psychometric superiority, but in extending contextual representation of postpartum stress experiences within collectivistic maternal settings.

One limitation of the present study is that the initial item-generation process was primarily informed by multidisciplinary expert perspectives and did not formally incorporate direct qualitative interviews with postpartum mothers. Although expert involvement strengthened the conceptual, clinical, cultural, and psychometric foundation of the instrument, expert-informed conceptualization may differ from the direct lived experiences and subjective stress perceptions of postpartum mothers. Future studies are recommended to integrate qualitative exploration with postpartum mothers during item development and contextual validation processes to further strengthen experiential and contextual representation of postpartum stress.

## 5 Limitations and future research

This study has several limitations that should be considered when interpreting the findings. First, the participants were limited to early postpartum mothers within a specific population context, which restricts the generalizability of the results to broader and cross-cultural populations. Second, the use of a cross-sectional design captures postpartum stress at a single point in time, whereas maternal stress experiences are dynamic and may change throughout the postpartum period. Third, data collection relied on self-report measures, which may introduce subjective bias in participants’ responses. In addition, this study focused primarily on examining construct validity and reliability without assessing predictive validity in relation to maternal mental health outcomes and infant well-being.

Future research is therefore recommended to conduct cross-cultural validation studies to ensure the applicability of the scale across diverse populations. Longitudinal designs are also needed to capture changes in postpartum stress over time and to better understand its developmental trajectory. Furthermore, future studies should examine predictive validity by investigating the relationship between postpartum stress and key outcomes such as postpartum depression, anxiety, maternal functioning, and infant development. It is also recommended to test the sensitivity of the scale in detecting changes following clinical or psychosocial interventions. In addition, integrating this instrument into digital health platforms or maternal care systems could enhance early screening and monitoring of postpartum stress in real-world settings. Expanding the use of the scale to different subpopulations, such as adolescent mothers or mothers with high-risk pregnancies, would further strengthen its applicability and practical value.

The findings of the present study should be interpreted within the sociocultural context of the study population. Postpartum experiences among Indonesian mothers are influenced by collectivistic family dynamics, traditional postpartum practices, breastfeeding expectations, and culturally shaped maternal role responsibilities, which may differ across settings and populations. Therefore, although the developed instrument demonstrated satisfactory psychometric performance within the present sample, broader applicability and generalizability of the scale to other postpartum populations should be interpreted cautiously. Future studies are recommended to conduct multicenter validation and cross-cultural psychometric evaluation in more diverse maternal populations.

Postpartum experiences among Indonesian mothers are strongly influenced by collectivistic family structures, traditional postpartum beliefs and practices, breastfeeding-related expectations, intergenerational caregiving involvement, and culturally shaped maternal role responsibilities. Therefore, the present instrument was developed to integrate psychological, physical, social, cultural, and support-related postpartum stress dimensions within a culturally contextualized psychometric framework relevant to Indonesian maternal settings.

## 6 Conclusion

This study successfully developed and evaluated the psychometric properties of a postpartum stress scale that demonstrates strong validity and reliability. The findings indicate that postpartum stress is a multidimensional construct consisting of four main factors that collectively represent the emotional distress experienced by mothers during the early postpartum period. The results of Exploratory and Confirmatory Factor Analysis demonstrate that the measurement model exhibits an adequate level of fit, supported by satisfactory construct reliability and convergent validity. These findings suggest that the developed scale has strong potential as a sensitive measurement tool for identifying postpartum stress levels in mothers. Consequently, the instrument can be utilized for early screening, clinical assessment, midwifery practice, and maternal health research, contributing to improved detection and management of postpartum psychological well-being.

## Data Availability

The raw data supporting the conclusions of this article will be made available by the authors, without undue reservation.
